# Quantitative Evaluation of Task-Induced Neurological Outcome after Stroke

**DOI:** 10.3390/brainsci11070900

**Published:** 2021-07-07

**Authors:** Iqram Hussain, Se-Jin Park

**Affiliations:** 1 Center for Medical Convergence Metrology, Korea Research Institute of Standards and Science, Daejeon 34113, Korea; iqram@ust.ac.kr; 2 Department of KSB (Knowledge-Converged Super Brain) Convergence Research, Electronics and Telecommunication Research Institute, Daejeon 34129, Korea; 3 Department of Medical Physics, University of Science & Technology, Daejeon 34113, Korea; 4 AI Research Group, Sewon Intelligence, Ltd., Seoul 04512, Korea

**Keywords:** electroencephalography, stroke, neuroscience, machine-learning, neurological workload

## Abstract

Electroencephalography (EEG) can access ischemic stroke-derived cortical impairment and is believed to be a prospective predictive method for acute stroke prognostics, neurological outcome, and post-stroke rehabilitation management. This study aims to quantify EEG features to understand task-induced neurological declines due to stroke and evaluate the biomarkers to distinguish the ischemic stroke group and the healthy adult group. We investigated forty-eight stroke patients (average age 72.2 years, 62% male) admitted to the rehabilitation center and seventy-five healthy adults (average age 77 years, 31% male) with no history of known neurological diseases. EEG was recorded through frontal, central, temporal, and occipital cortical electrodes (Fz, C1, C2, T7, T8, Oz) using wireless EEG devices and a newly developed data acquisition platform within three months after the appearance of symptoms of ischemic stroke (clinically confirmed). Continuous EEG data were recorded during the consecutive resting, motor (walking and working activities), and cognitive reading tasks. The statistical results showed that alpha, theta, and delta activities are biomarkers classifying the stroke patients and the healthy adults in the motor and cognitive states. DAR and DTR of the stroke group differed significantly from those of the healthy control group during the resting, motor, and cognitive tasks. Using the machine-learning approach, the C5.0 model showed 78% accuracy for the resting state, 89% accuracy in the functional motor walking condition, 84% accuracy in the working condition, and 85% accuracy in the cognitive reading state for classification the stroke group and the control group. This study is expected to be helpful for post-stroke treatment and post-stroke recovery.

## 1. Introduction

Acute ischemic stroke and intracerebral hemorrhage are the leading causes of neurological disorders among the elderly population, and they affect millions of people with neurological deficits, physical disabilities, and dependent lifestyles [1,2]. An ischemic lesion affects the functional network architecture of cortical areas and hampers functional motor cognitive outcomes [3,4,5]. Neurological impairment due to stroke contributes to disability, poor functional improvement, and lower quality of life. In addition, the cognitive deficit can reduce the usefulness of post-stroke rehabilitation and vastly increase the risk for psychological disorders such as depression and anxiety. Moreover, the economic burden of the post-stroke treatment of patients with physiological impairment is significantly greater than those without. Precise identification of aspects that predict cognitive and functional outcomes is needed for making clinical decisions, setting feasible targets and plans for rehabilitation, and directing patients accordingly.

Tracking cortical activity is essential for identifying cognitive impairment due to stroke [3,6]. As an ischemic, or hemorrhage, stroke happens due to the breakdown of blood cells, it obstructs the flow of oxygen towards the brain cells of the stroke-affected lesion part and lets the brain tissues expire. The neuro-electrical activity of the representative cortical lobe is disturbed due to this damage to brain cells and destabilizes the overall neural system. Computed tomography (CT) and magnetic resonance imaging (MRI) are usually utilized as detailed assessment techniques to recognize brain anatomy and the severity of neural deficits resulting from stroke (thrombosis or hemorrhage) [2]. Nevertheless, the low temporal resolution and the higher economic burden limit the usage of those imaging methods.

Functional motor and cognitive deficits are usual and persistent consequences of stroke and a significant factor responsible for physical dysfunction, slow physiological recovery, and a worsened post-stroke lifestyle. Conventional mental and neurological evaluations can’t be conducted immediately after stroke due to the medical aspects (e.g., fluctuating levels of arousal, pain, confusion, tiredness) and functional obstacles (e.g., sensory, linguistic, motor shortfalls) that hamper the patients’ capability to approach physiological examinations [7].

Electroencephalography (EEG) is a non-invasive imaging method with a low spatial resolution but high temporal resolution and is sensitive in understanding irregularities of cerebral rhythms caused by stroke. The neurological outcomes can be easily tracked through EEG. Due to its non-invasive nature, EEG is considered as an alternative to traditional clinical cognitive tests [8]. The physiological signal can be an effective tool of a real-time physiological monitoring system for early prognostics in a daily-life setup [4,6,9,10,11,12]. During the rehabilitation of stroke patients, EEG changes can help to track the post-stroke recovery in daily life and clinical setup. Plentiful EEG studies were reported to investigate EEG in medical and wellness settings to explore the correlation between the EEG features and the neurological outcomes after ischemic stroke [5,7,13,14,15,16,17,18]. The rise of slow-wave delta activity and the decline of fast-wave activity were found as predictive markers of reduced functional outcomes in the lesion area, and the nonexistence of these phenomena was reported as excellent mental outcomes [3,19]. The role of these frequency bands can be characterized using the delta–alpha ratio (DAR), which computes the relative measure of the delta wave and the alpha wave.

We hypothesized that variations in the electrical activity of the brain due to the neural deficit would be acknowledged by the EEG measurements. The feature extraction using signal processing and dimensionality reduction, feature ranking, and machine-learning methods would be a trustworthy technique for understanding post-stroke cognitive impairment and recovery of stroke patients.

We explored the neuro-electrical activity of the stroke group and the control group through EEG while resting and performing motor and cognitive tasks. Our objective is to quantify EEG features for understanding task-induced neural changes because of stroke-derived physiological impairment, and to identify the biomarkers to distinguish the stroke patients and healthy adults.

The rest of this study was organized into five sections. Section 2 described the experimental protocol and the methodology of the study. The results were demonstrated in Section 3, followed by the discussion. Lastly, the conclusions were narrated in Section 5.

## 2. Materials and Methods

### 2.1. Data Acquisition Platform

**Figure 1:**
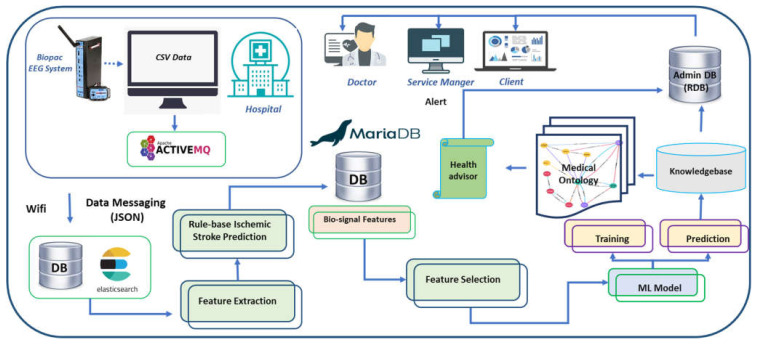
The architecture of the data acquisition framework of the stroke patient monitoring system. Wireless EEG device sends the brainwave of the patients to a nearby computer through Bluetooth communication. EEG data is initially stored in CSV format and then converted to JSON format. An API transfers JSON format EEG HL7 V2 messages to the webserver using ActiveMQ. Feature extraction algorithms extract the neurological features, and rule-based disease prediction labels the data with the possible diseases. The machine-learning model learns and predicts the neural impairment status. Hospitals and clients can monitor the signals and access the health advice through their dedicated portals.

A physiological data acquisition platform was developed for prognostics of diseases and management of treatment for post-stroke patients. As demonstrated in Figure 1, the EEG Data Acquisition Platform consists of wireless EEG sensors, the application programming interface (API), the networking module, the big data-based cloud storage and processing, and the data visualization system. The raw-data API sent neuro-electrical health level 7 (HL7 V2) messages, created as the protocol of the standards of HL7 International, to the Elasticsearch database (DB) of the webserver through the Wi-Fi or LTE network. Relevant brainwave features, such as spectral power measures, were extracted using the feature extraction algorithms. The rule-based disease prediction algorithms were based on the acute illness predictive features, such as the asymmetric characteristics and the ratios of spectral power, and labelled brainwave features with possible diseases. Feature selection selects the statistically significant features for machine-learning analysis. The selected neuro-electrical features are fed to the machine-learning model for training and testing machine-learning classification models. The medical ontology can suggest possible disease prediction as assistance for managing the post-stroke treatment. All processed data can be visualized in dedicated monitors for patients, service providers, and assigned medical doctors. 

### 2.2. Study Design

**Figure 2:**
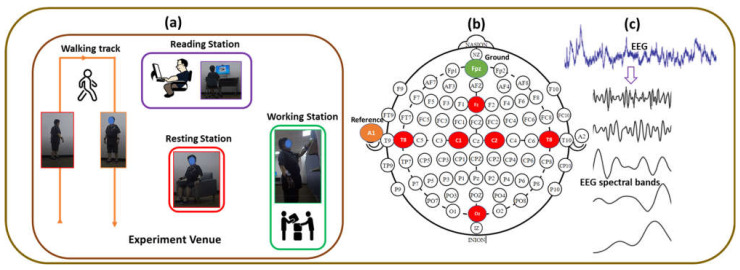
EEG Signal Processing and electrode positions and layout. (**a**) Experimental scenario, (**b**) six-channel EEG, reference and ground electrodes position based on Standard 10–20 EEG system, (**c**) EEG Signal Processing.

The study was conducted according to protocol approved by the Institutional Review Board of Korea Research Institute of Standards and Science, Daejeon, South Korea. Before the start of the experiment, the experimental scenario was explained to the participants. EEG electrodes were attached to the participants and connected to a wireless EEG device. As shown in Figure 2a, the study consists of various activities, such as resting, and motor and cognitive workload. The resting task consists of resting while lying down on the bed and resting while sitting on the sofa. The motor task includes walking following a line and moving a bottle up and down between vertical shelves. The cognitive task was composed of reading literature not previously familiar to participants. EEG data were continuously recorded throughout the entire experiment. At first, the participants were instructed to rest on the bed for three min, followed by the line-following walking; then, they were asked to move bottles over vertical racks, followed by resting on a chair. Finally, they were allowed to read the article as a cognitive task. Room temperature was maintained at 24 °C, and relative humidity was 40%.

### 2.3. Demographics of Participants

Forty-eight stroke patients and 75 healthy adults were recruited for this study. The target group consists of 48 stroke patients (mean age: 72.2 ± 5.6 years, 62% male). The control group comprised 75 healthy adults (mean age 77 years, 31% male). Both the stroke and control groups were selected within the same age range to decrease age-linked physiological pattern variation. The participants comprised patients referred to the Stroke Rehabilitation Center, Chungnam National University Hospital, Daejeon, South Korea. Ischemic stroke happenings of the patients were confirmed through MRI scans or CT (clinically). The severity of stroke patients can be characterized by the National Institute of Health Stroke Scale (NIHSS) score. The highest severity score is marked as 42, and the lowest is labeled as 0. Out of the total of 48 stroke patients studied in this study, seven patients experienced a minor stroke (NIHSS: 1–4), ten patients were identified with a moderate stroke (NIHSS: 5–15), 13 patients diagnosed with a moderate-to-severe stroke (NIHSS: 16–20), and eighteen patients had a severe stroke (NIHSS: 21–42). The control group comprises healthy adults without any history of ischemic or hemorrhagic stroke or underlying known neurologic disorders.

### 2.4. EEG Data Acquisition

In this study, the EEG was recorded as the representative signal of the neural system of the brain. Six-channel EEG data were acquired using a Biopac MP 160 Module (Biopac Systems Inc., Goleta, CA, USA) with the AcqKnowledge ver 5.0 software. The electrical activity of the cerebral cortex was continuously recorded using a wireless Biopac BioNomadix EEG amplifier (EEG2-4.3) at a sampling rate of 1000 Hz. The amplifier captured the signal via reusable gold-plated 10 mm diameter cup electrodes (EL160) attached to the scalp with a conductive paste (Elefix, Nihon Kohden, Japan). In this study, EEG data were taken on the Fz, Oz, C1, C2, T7, and T8 positions, according to the international 10–20 EEG system, as shown in Figure 2b. Fz is a characteristic electrode of the frontal lobe; Oz is a characteristic electrode of the occipital lobe; C1 and C2 exist in the central lobe; and T7 and T8 are the representative positions of the temporal lobe. EEG recording was performed in a unipolar setup. The reference electrode was placed in A1, the mastoid bone close to the ear, and the ground electrode was placed in the Fpz position. In addition, one-channel vertical electrooculogram (VEOG) was recorded for filtering eye blinks, and one-channel chin electromyogram (EMG) was recorded to eliminate the muscular artifact. In the case of the stroke population, EEG data were recorded no later than three months after the diagnosis of ischemic stroke. Participants were recommended to avoid coffee or alcohol before the test. EEG data were recorded during the resting state, walking state, working state, and reading state. The resting-state EEG was recorded in an eye-closed sitting state. In general, EEG becomes affected by eye-blink and muscular artifacts during ambulatory and active conditions, such as walking, working, and reading tasks. The EOG and chin EMG were utilized for denoising eye-blink and muscular artifacts. A neurologist scored the EEG recordings ranging from level “0” to level “3” based on task-induced mental workload. 

### 2.5. Pre-Processing

Firstly, 60 Hz AC noise was filtered out from the raw EEG signal. Independent component analysis (ICA) was utilized to remove ocular and muscular artifacts in the EEG. We used the FastICA algorithms for denoising the EEG signal [20]. ICA utilized EOG and EMG recordings to isolate the EEG waveform from the eye-blink and muscular artifacts. The EEG waveform was filtered within the 0.5–44 Hz frequency range using a band-pass filter. AcqKnowledge ver 5.0 software (Biopac Systems Inc., Goleta, CA, USA) was used for pre-processing and feature extraction of EEG data.

### 2.6. Feature Extraction

EEG frequency-specific waveforms, such as delta (δ), theta (θ), alpha (α), beta (β), and gamma (γ) oscillations were extracted from the artifact-free EEG signal as shown in Figure 2c. EEG spectral waves were analyzed using the Welch periodogram estimation method [21]. The Fast Fourier Transforms (FFT) method was applied on artifact-free EEG signals with a 10% hamming window to determine the power spectral density (PSD) of the frequency components in the EEG signal. EEG signal was divided into fixed-width time epochs of 10 s each. Mean power, median frequency, mean frequency, and spectral edge features of frequency domains were extracted over the frequency ranges. A few key features were described in detail in the subsections.

#### 2.6.1. EEG Relative Power of Frequency Bands

EEG relative power (RP) was calculated through Fast Fourier transforms (FFT) performed on EEG signal with a 10% hamming window. We extracted absolute power in the following spectral frequency bands: the delta (δ) waveform was characterized in the range of 0.5–4.0 Hz, the theta (θ) wave in a range of 4.0–8.0 Hz, the alpha (α) wave in the range of 8.0–13.0 Hz, the beta (β) band in the range of 13.0–30 Hz, and the gamma (γ) band in the range of 30.0–44 Hz. All EEG power features were measured each with a 10 s epoch. Relative band power was defined as Equation (1).

(1) $$p_{j}=\frac{P_{j}}{\sum_{j=1}^{q}P_{j}}$$

where P_j_ is absolute spectral power density with frequency j (with j =1,2, ..., q), and q is the frequency ranging 0.5–4 Hz, 4–8 Hz, 8–13 Hz, and 13–30 Hz. The changes in EEG power on motor and cognitive states relative to the resting state would help understand the characteristics of neurological phase change for the stroke patients and the healthy control participants. At first, the average EEG power of each band was derived in the resting state. The average resting EEG band power, ${\bar{rp}}_{r}$ was calculated as Equation (2).

(2) $${\bar{rp}}_{r}=\frac{\sum_{k=1}^{n}rp_{k}}{n}$$

where rp_k_ is the relative power of each epoch. Then, the change of EEG power during active states (motor task and cognitive task) relative to the resting state (baseline)$,\;\Delta p_{m}$, was calculated as Equation (3).

(3) $$\Delta p_{m}=\frac{\left(p-{\bar{rp}}_{r}\right)}{{\bar{rp}}_{r}}*100\%$$

where m is the level of task, such as resting, walking, working, and reading.

#### 2.6.2. Pairwise Derived Brain Symmetry Index

The pairwise derived brain symmetry index (pdBSI) is a biomarker for hemispheric stroke and is calculated as suggested by Sheoralpanday [22] and Van putten [23]. Hemispheric stroke results in a lack of cortical power balance between the two hemispheres. As pdBSI is the indicator of hemispheric power asymmetry, it may be used for early stroke prediction [3,13]. pdBSI ranges between zero (no asymmetry) and one (total asymmetry). The pdBSI was defined in Equation (4).

(4) $$pdBSI=\frac{1}{pq}\overset{q}{\underset{j=1}{\sum }}\overset{p}{\underset{i=1}{\sum }}\left|\frac{Rt_{ij}-Lt_{ij}}{Rt_{ij}+Lt_{ij}}\right|$$

with Rt_ij_ and Lt_ij_ being the power spectral density of the waveform of a right and a left node of a homologous EEG channel pair (with i = 1,2, ..., p) at frequency j (with j =1,2, ..., q). For this study, p was limited to 2, and pdBSI was measured for the frequency bands (q) 0.5–4 Hz, 4–8 Hz, 8–13 Hz, and 13–30 Hz.

#### 2.6.3. DTR, DTABR, and DAR

Delta–theta ratio (DTR) is described as the fraction of delta wave power relative to theta wave power. (Delta+Theta)/(Alpha+Beta) ratio (DTABR) is identified as the relative sum of slow-wave (delta rhythm and theta rhythm) power to fast-oscillating wave (alpha rhythm and beta rhythm) power. Delta–alpha ratio (DAR) is defined as the fraction of delta wave power relative to alpha wave power. EEG delta wave power ratios (DAR, DTR, and DTABR) were effective biomarkers of cognitive and neurological changes due to stroke [7].

(5) $$DAR=\frac{p_{j=\delta }}{p_{j=\alpha }}$$

(6) $$DTR=\frac{p_{j=\delta }}{p_{j=\theta }}$$

where j is the EEG frequency-specific rhythms, delta wave (δ) ranges 0.5–4.0 Hz, theta wave (θ) ranges 4.0–8.0 Hz, and alpha wave (α) ranges 8.0–13.0 Hz.

#### 2.6.4. Spectral Band Power Asymmetry

Spectral Band Power Asymmetry is defined as the relative difference of neuro-electrical power of EEG rhythms among the right and left cortical hemispheres. It was reported that EEG spectral band asymmetries were associated with stroke, depression, epilepsy, apnea, and so on [3,24].

(7) $$Spectral\;Band\;power\;asymmetry,=\frac{1}{p}\overset{p}{\underset{i=1}{\sum }}\left|\frac{Rt_{i}-Lt_{i}}{Rt_{i}+Lt_{i}}\right|$$

### 2.7. Features Selection

Feature selection, or feature ranking, reduces data processing time and memory requirements so that machine-learning algorithms can deal with only the essential predictors. For the selection of the best-ranked features, feature importance was computed. Feature importance was evaluated through Pearson’s chi-square test. Features were ranked through screening, ranking, and selection processes. We screened out the features with constant and missing values in the early steps. The predictor’s importance was determined based on how effectively each variable independently forecasts the target class. Feature importance greater than 95% was the criteria in the feature selection process.

### 2.8. Classification Algorithms

We explored the machine-learning algorithms for automatic discrimination of the neural features of the ischemic stroke group and the healthy adult group during the resting, walking, working, and cognitive reading tasks. Seventy percent of EEG feature data was labeled as the training dataset, and thirty percent of EEG features were kept as the testing dataset. C5.0, Support Vector Machine (SVM), logistic regression, and Random Trees models have been implemented to discriminate neurological features of acute patients and healthy adults. The C5.0 model is a supervised data mining tool used to build decision trees using a divide-and-conquer method. Support Vector Machines (SVM) map data in a high-dimensional hyperplane so that features can be classified by creating the marginal line using Radial Basis Function (RBF)-kernel functions. We trained the SVM model and performed k-fold cross-validation (k = 10). Random Trees is an ensemble learning algorithm generating classifications and regression trees with decision rules to understand the prediction of the trees.

### 2.9. Data Analysis

Several EEG measures, such as relative power of frequency bands, mean power of spectral components, mean frequency and median frequency of EEG rhythms, peak frequency, and spectral edge components over the standard EEG frequency ranges, were analyzed using the statistical and machine-learning approach. Methods of statistical analysis consisted of descriptive statistics and hypothesis tests, such as an independent-samples t-test. SPSS 24 software (IBM, Armonk, New York, NY, USA) was utilized for statistical investigation. We evaluated the feature importance of EEG features based on their individual prediction accuracy and ranked them using the feature selection technique. We assessed the feature importance described as (1-*p*) using Pearson’s chi-square method, where *p* is the probability of association of the feature with the stroke group or control group. We partitioned the selected feature datasets into two categories: the training dataset and the testing dataset. The training dataset was forwarded to the machine-learning algorithms to train the classification models, which were later utilized for predicting the testing datasets. We used IBM SPSS Modeler 18 software (IBM, Armonk, New York, NY, USA) for machine-learning analyses.

## 3. Results

### 3.1. Statistical Investigation

We investigated the brainwave features of the ischemic stroke group and control group using descriptive statistics to explore the task-induced changes in the electrical activity of the brain. We also conducted hypothesis tests, such as the independent-samples t-test, to evaluate the statistical significance of variations of EEG features for two groups. This test performed Levene’s test to measure equality of variances and a t-test for the equality of means. The statistical significance was characterized as a *p*-value of less than 0.05.

#### 3.1.1. Association of Spectral Power with Stroke and Mental Workload

**Figure 3:**
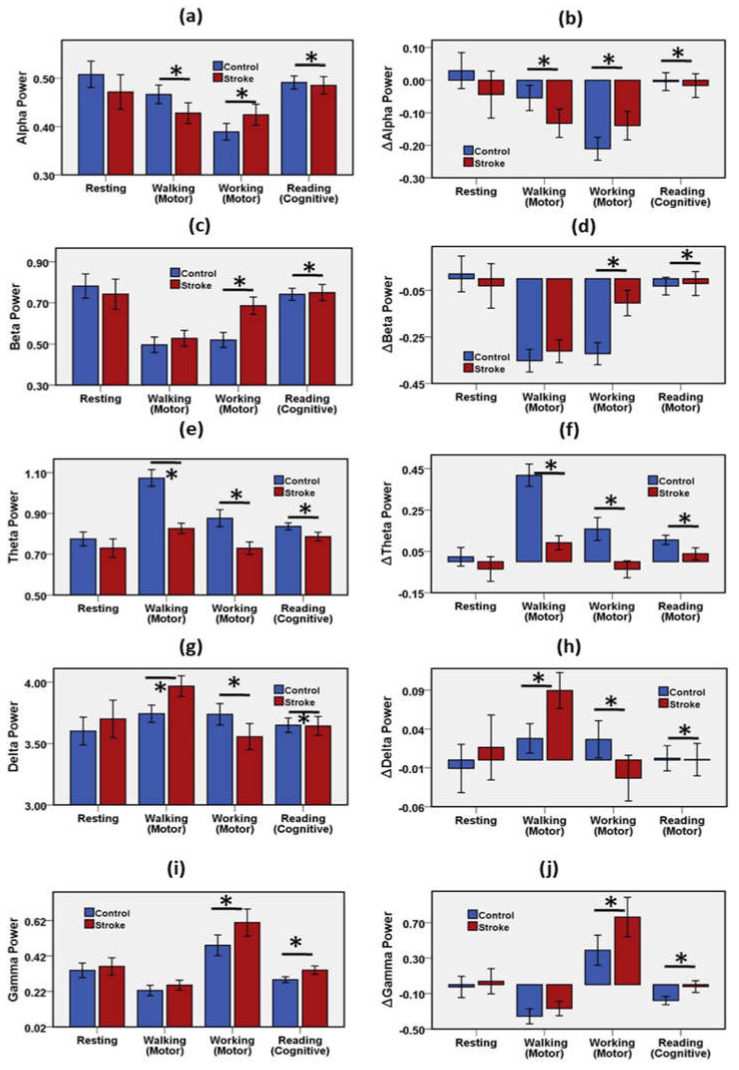
Mean and error bar of (**a**) RP Alpha, (**b**) change of Alpha relative to baseline, (**c**) RP Beta, (**d**) change of Beta relative to baseline, (**e**) RP Theta, (**f**) change of Theta relative to baseline, (**g**) RP Delta, (**h**) change of Delta relative to baseline, (**i**) RP Gamma, (**j**) change of Gamma relative to the baseline of the stroke group and the control group during resting, motor, and cognitive tasks. Error bar shows 95% confidence interval. * (*p* < 0.05) indicates a significant difference. Resting state was considered as baseline. RP = Relative Power.

EEG spectral power of the stroke and healthy control groups were evaluated in various activities: resting, walking, working, and reading. Those activities varied based on neurological workload. Figure 3 shows the mean and error bar with a 95% confidence interval of EEG spectral bands’ RP during the resting, motor, and cognitive tasks. Table S1 presents the results of the statistical analysis of the EEG spectral features for the control group and the stroke group during the resting, motor, and cognitive tasks. 

Alpha was dominant in the resting state. Alpha power of the stroke group was lower than the control group in the resting and walking tasks, similarly in the cognitive state, such as reading, task. Resting alpha mean RP was 0.51 in the control group and 0.47 in the stroke group. In the walking state, alpha decreased by −5% in the control group and −13% in the stroke group relative to the resting state. In the working state, alpha decreased by −21% in the control group and −14% in the stroke group relative to the resting state. In the reading state, alpha decreased slightly relative to the resting state. Alpha power of the stroke group was a significantly important biomarker to discriminate the stroke group and the control group in the brain motor control (walking and working) and cognitive (reading) state. 

Beta was leading in the resting and reading state. Beta power of the stroke group was higher than the control group in the resting mode, lower in the motor states, and similar in the cognitive state. Resting beta mean RP was 0.78 in the control group and 0.74 in the stroke group. In the walking state, beta decreased by −35% in the control group and −31% in the stroke group relative to the resting state. In the working state, beta decreased by −32% in the control group and −10% in the stroke group relative to the resting state. In the reading state, beta decreased by −3% in the control group and −2% in the stroke group relative to the resting state. Beta power of the stroke group was a significantly important biomarker to distinguish the stroke group and the control group in the working and reading states.

Theta power of the stroke group was higher than the control group in all states: resting, motor, and cognitive. Resting theta mean RP was 0.77 in the control group and 0.73 in the stroke group. In the walking state, theta increased by 42% in the control group and 9% in the stroke group relative to the resting state. In the working condition, theta increased by 16% in the control group and decreased by −4% in the stroke group relative to the resting state. In the reading state, theta increased by 11% in the control group and 4% in the stroke group relative to the resting state. Theta power of the stroke group was a significantly important biomarker to classify the stroke group and the control group in the walking, working, and reading conditions.

Resting delta mean RP was 3.60 in the control group and 3.70 in the stroke group. In the walking state, delta increased by 3% in the control group and 9% in the stroke group relative to the resting state. In the working state, delta increased by 3% in the control group and decreased by −2% in the stroke group relative to the resting state. Delta rose slightly in the reading state relative to the resting state. Delta power of the stroke group was a significantly important biomarker to differentiate the stroke group and the control group in the walking, working and reading conditions.

The mean value of resting relative gamma power was 0.33 in the control group and 0.36 in the stroke group. In the walking state, gamma decreased by −36% in the control group and −27% in the stroke group relative to the resting state. In the working state, gamma increased by 39% in the control group and 76% in the stroke group relative to the resting state. In the cognitive state, gamma decreased by −18% in the control group and −2% in the stroke group relative to the resting state. The gamma power of the stroke group was significantly different from the control group in the working and reading states.

#### 3.1.2. Distribution of Task-Induced Spectral Power among Cortical Lobes

**Figure 4:**
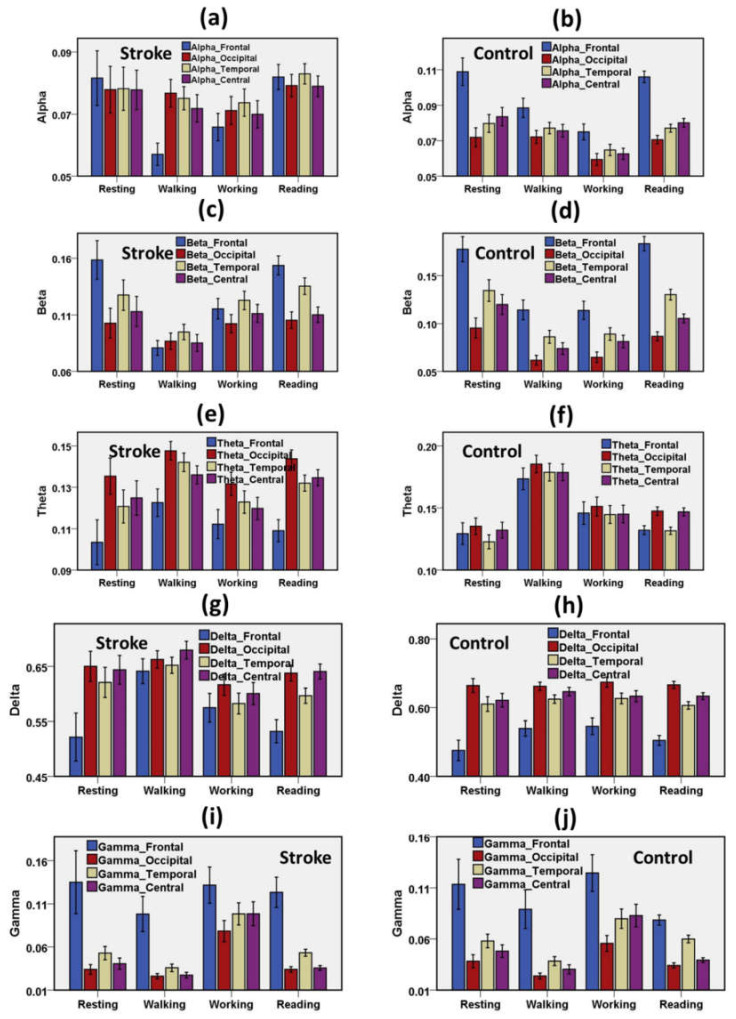
Distribution of spectral waves on the cortical regions (frontal lobe, central lobe, temporal lobe, occipital lobe) during resting, motor, and cognitive tasks. (**a**) RP Alpha of stroke group (**b**) RP Alpha of the control group, (**c**) RP Beta of stroke group (**d**) RP Beta of the control group, (**e**) RP Theta of stroke group (**f**) RP Theta of the control group, (**g**) RP Delta of stroke group (**h**) RP Delta of the control group, (**i**) RP Gamma of stroke group (**j**) RP Gamma of the control group. Error bar shows 95% confidence interval. RP = Relative Power.

Frequency-specific EEG power measures of the stroke and healthy control groups were assessed through lobe-specific electrodes during resting, motor, and cognitive activities. The distribution of task-induced frequency-specific power measures among cortical lobes was demonstrated in Figure 4. Alpha was higher in the frontal lobe compared with other lobes during the resting and cognitive states for the stroke group and the healthy control group. Alpha power is lowest in the frontal lobe during walking states for stroke patients. On the other hand, the lowest alpha power was observed in the occipital lobe during the working tasks for the healthy control group. The beta wave was dominant in the frontal lobe during resting and cognitive states for the stroke group and the healthy control group. Weak beta power was reported in the motor states for the stroke patients. On the contrary, beta power was weakest in the occipital lobes for the healthy control group during the motor tasks. Strong theta waves were observed in the occipital lobe during resting, motor, and cognitive states for the stroke group, but the higher theta was found only during the walking task in the entire cortex for the healthy control group. The delta wave was weakest in the frontal lobe and strongest in the occipital lobe during resting, motor, and cognitive states for the healthy adults. The delta wave was dominant in the occipital, temporal, and central brain lobes during all tasks for the stroke group. Higher delta power was an indicative biomarker of ischemic stroke. The gamma wave was most substantial in the frontal cortex during every task for the stroke group and the healthy control group.

#### 3.1.3. Association of Spectral Power Asymmetry with Stroke Recovery

**Figure 5:**
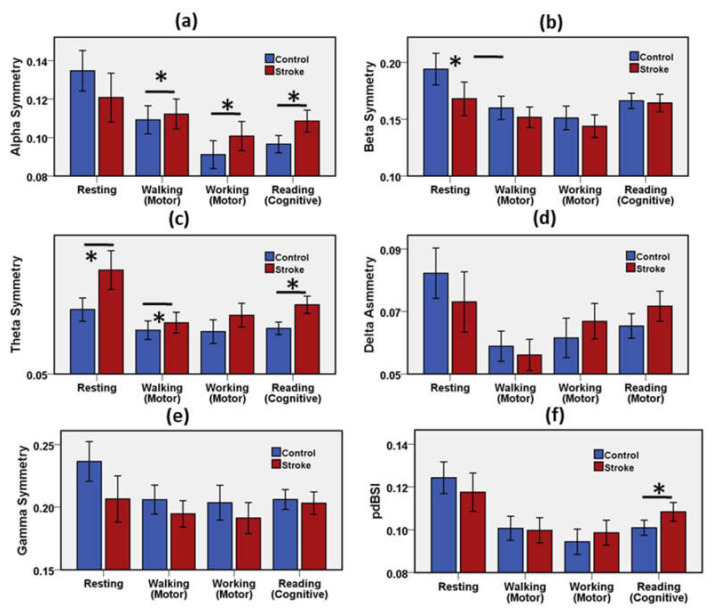
Mean and error bar of (**a**) Alpha asymmetry, (**b**) Beta asymmetry (**c**) Theta asymmetry, (**d**) Delta asymmetry (**e**) Gamma asymmetry, (**f**) pdBSI of the stroke group and the control group during resting, motor, and cognitive tasks. Error bar shows 95% confidence interval. * (*p* < 0.05) indicates a significant difference.

The symmetric characteristic of EEG spectral power of the left and right cortex was explored in the stroke group and the control group. EEG measured in the C1 and T7 electrodes was representative of the left cortex, and the EEG in the C2 and the T8 was representative of the right cortex. The difference between the two cortexes provides the asymmetric measure. Figure 5 shows the mean and error bar with a 95% confidence interval of the asymmetries of the RP of the EEG spectral bands. Table S2 presents the results of the statistical analysis of the asymmetry of spectral components for the control group and the stroke group during the resting, motor, and cognitive tasks.

Alpha asymmetry was significantly different in the walking, working, and reading conditions. Resting alpha symmetry showed no association with a change in EEG spectrum due to ischemic stroke. Beta asymmetry was significantly different in the resting condition. Beta asymmetry indicated no association with change in EEG spectrum due to ischemic stroke in motor and cognitive tasks. Theta asymmetry was significantly different in the resting, walking, and reading tasks. Theta symmetry had no association with a change in EEG spectrum due to stroke in working task. Asymmetric characteristics of delta and gamma waves revealed no significant difference in any neurological state to distinguish the neural impairment in the stroke group relative to the control group. The pdBSI showed significant variation during only the cognitive task.

#### 3.1.4. DTR, DTABR, and DAR as Biomarkers for Stroke Prediction

DTR, DTABR, and DAR were explored during the motor task, cognitive workload, and resting mode to understand their relationship with neural impairment due to ischemic stroke. Results of the statistical analysis of the DTR, DTABR, and DAR for the control group and the stroke group during the resting, motor, and cognitive tasks are presented in Table S3.

**Figure 6:**
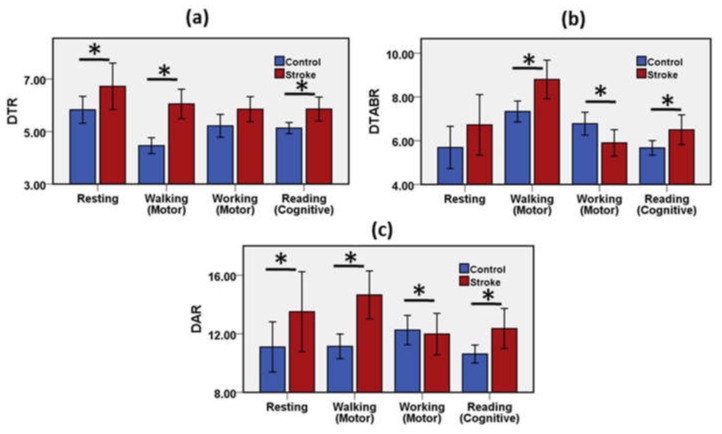
Mean and error bar of (**a**) DTR, (**b**) DTABR, (**c**) DAR of the stroke group and the control group during resting, motor, and cognitive tasks. Error bar shows 95% confidence interval. * (*p* < 0.05) indicates a significant difference.

As displayed in Figure 6a, DTR showed a significant statistical difference between the ischemic stroke patients and the healthy adults during resting, walking, and reading activities. The mean value of DTR was 5.83 in the control group and 6.72 in the stroke group while resting. In functional motor tasks, such as walking, the mean value of DTR was 4.46 in the control group and 6.05 in the stroke group, whereas the mean value of DTR was 5.22 in the control group and 3.63 in the stroke group during the functional motor movement, such as working, activity. The mean value of DTR was 5.13 in the control group and 5.86 in the stroke group throughout the cognitive task. DTR was observed as a statistically significant important biomarker to discriminate the stroke and the control group during the resting state, functional motor task, and mental workloads.

As demonstrated in Figure 6b, DTABR showed a significant statistical difference between the ischemic stroke patients and the healthy adults during functional motor (walking, working) and cognitive reading activities. Resting DTABR was 5.69 in the control group and 6.73 in the stroke group, and no significant difference in DTABR was observed between the stroke patients and the healthy control adults. The mean value of DTABR was 7.33 in the control group and 8.80 in the stroke group during the walking phase, whereas the mean value of DTABR was 6.78 in the control group and 5.90 in the stroke group during the working activity. The mean value of DTABR was 5.67 in the control group and 6.50 in the stroke group throughout the cognitive task. DTABR was observed as the biomarker to differentiate the stroke and the control group throughout the functional motor task and mental workload.

As presented in Figure 6c, resting DAR was 11.10 in the control group, 13.51 in the stroke group, and a significant difference in DAR was observed between the stroke patients and the healthy control adults. The mean value of DAR was 11.14 in the control group and 14.65 in the stroke group during the walking phase, whereas the mean value of DAR was 11.98 in the control group and 13.17 in the stroke group during the working activity. The mean value of DAR was 10.62 in the control group and 12.36 in the stroke group throughout the cognitive task. DAR was observed as the biomarker to classify the stroke and the control group in the functional motor task and mental workload setting.

#### 3.1.5. Variation of EEG Spectral Power with Mental Workload

**Figure 7:**
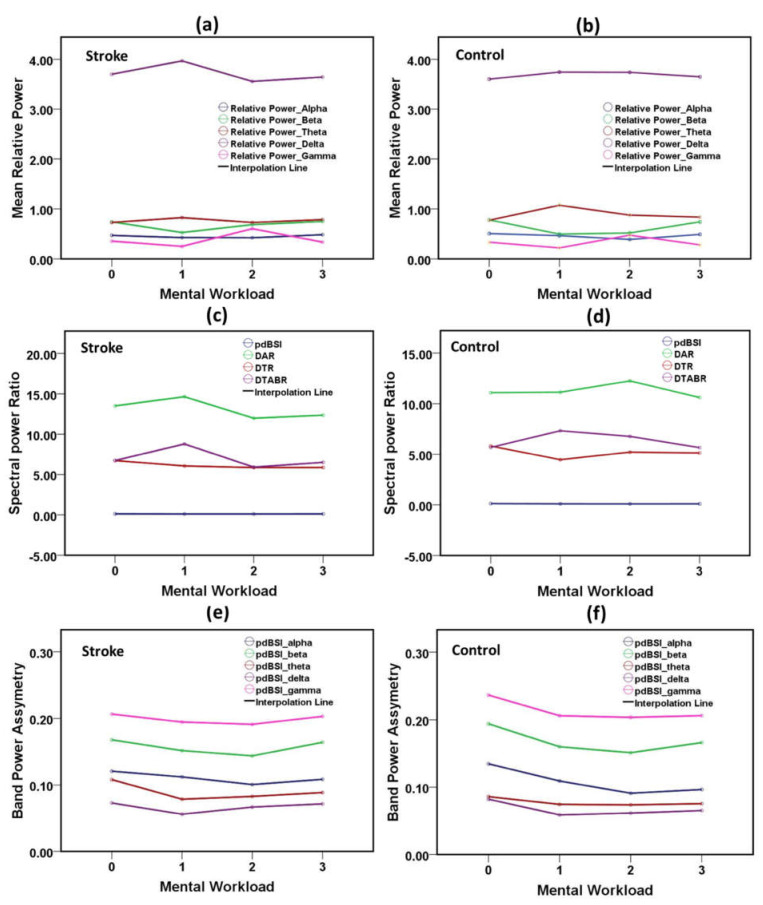
Variation of EEG spectral features, the ratios of power features and pdBSI band-specific components with mental workload. (**a**) Spectral RP with the mental workload for stroke group, (**b**) spectral RP with the mental workload for the control group, (**c**) frequency-band power ratios with the mental workload for stroke group, (**d**) frequency-band power ratios with the mental workload for the control group, (**e**) spectral components of pdBSI with the mental workload for stroke group, (**f**) spectral components of pdBSI with the mental workload for the control group.

EEG spectrum changes with the neurological outcomes due to mental workload. Resting was considered as zero (“0”), walking as one (“1”), working as two (“2”), and cognitive reading was scored as a level “3” mental workload. The variation of relative EEG spectral power components in various mental states of the stroke group and control group are displayed in Figure 7a,b. Delta is the dominant wave in spectral power. Delta was stable with the variations in the neural status of the healthy control group. In stroke patients, the delta fluctuated over the change of neurological load, and theta oscillations were higher in the neural motor states, such as walking and working. The change in DAR, DTR, DTABR, and pdBSI of the stroke patients and the healthy adults in various mental states are presented in Figure 7c,d. DAR was highest in the working task for the control group, whereas DAR is lowest in the stroke group during the working state. The band-specific pdBSI components of the stroke patients and the healthy adults in several mental states are demonstrated in Figure 7e,f. A sharp decline of theta power was observed from the resting state to the motor state in the stroke group. 

#### 3.1.6. Correlation of DAR, DTR with Mental Workload

The scatterplot and the regression line of delta power with theta power and alpha power were explored to understand the correlation of delta power with theta and alpha power during varied mental workloads (Resting, Walking, Working, Reading tasks), as shown in Figure S2.

In the stroke group, delta and alpha have strong negative correlations in the resting-state (correlation coefficient, r = −0.90), the walking state (r = −0.93), and the reading-state (r = −0.91), whereas a moderate negative correlation in the working condition (r = −0.51). 82% of the resting delta and alpha data variation were determined by this regression line (coefficient of determination, r^2^ = 0.82). The plotted regression lines described 86% of motor walking data, 82% of cognitive task data, and 26% of working state data. In the control group, the plotted regression lines described 68% of resting delta-alpha data (r^2^ = 0.68), 77% of functional motor walking data (r^2^ = 0.77), 38% of working state data (r^2^ = 0.38) and, 82% of cognitive task data (r^2^ = 0.82). The delta and alpha have strong negative correlations in the resting-state (r = −0.82), the walking state (r = −0.88), and the reading-state (r = −0.91), whereas a moderate negative correlation in the working state (r = −0.62).3.2. Machine-Learning Approach

### 3.2. Machine-Learning Approach

All EEG features with a feature importance of a *p*-value greater than 0.95 have been selected for the classification analysis. A total of 171 EEG features, having feature importance greater than 0.95, were selected beyond 244 brainwave features. The receiver operating characteristic (ROC) curve is the characteristic curve for the classification performance. The area under the curve (AUC) is a predictive performance feature and defined as the area under the ROC curve, ranging from 0 (zero) to 1 (one). The Gini is an alternative parameter to the AUC and is calculated as double of (AUC-1). The prediction performance parameters, such as accuracy (ACC), sensitivity (true positive rate), specificity (true negative rate), precision (positive predictive rate), negative predictive value, AUC, and Gini coefficient derived from the confusion matrix or error, were evaluated. The performance assessment parameters were calculated using the following standard formulae:

$$\mathrm{Sensitivity}=\frac{TP}{TP+FN}$$

$$\mathrm{Specificity}=\frac{TN}{TN+FP}$$

$$\mathrm{Precision}=\frac{TP}{TP+FP}$$

$$\mathrm{Negative}\;\mathrm{predictive}\;\mathrm{value}\;\left(\mathrm{NPV}\right)=\frac{TN}{TN+FN}$$

$$\mathrm{Accuracy}\left(\mathrm{ACC}\right)=\frac{TN+TP}{TN+TP+FN+FP}$$

where TP stands for the true positive, TN means the true negative, FP stands for the false positive, and FN means the false negative.

#### 3.2.1. Prediction of Stroke Group and Control Group through Classification Models

**Figure 8:**
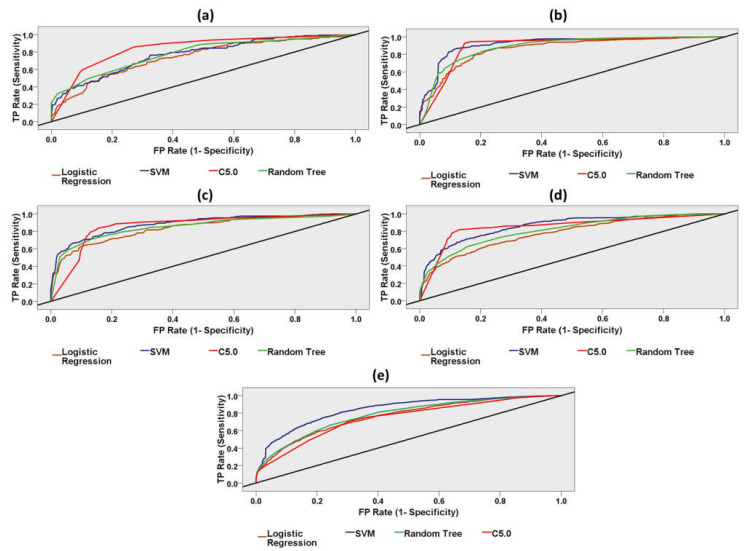
Receiver Operating Characteristic (ROC) curves for four different machine-learning models (Support Vector Machine, Random Trees, Logistic Regression, C5.0). (**a**) ROC curve for resting dataset, (**b**) ROC curve for walking dataset, (**c**) ROC curve for working dataset, (**d**) ROC curve for reading dataset, (**e**) ROC curve for task-independent entire dataset. The area under the ROC curve (AUC) is an indicator of prediction accuracy. C5.0 classified the cognitive reading dataset with the highest AUC (86%) and highest accuracy (ACC: 85%). The diagonal black line is the reference line.

As shown in Table 1, Table 2, Table 3, Table 4 and Table 5, we investigated machine-learning models to predict the neural impairment of ischemic stroke patients relative to the healthy control group using a resting, walking, working, reading, and task-independent entire dataset. In the resting dataset, the C5.0 algorithm classified the stroke patients and the healthy adults with 78% accuracy (AUC: 0.84), the highest precision (79%), and the highest specificity (90%). In the functional motor task (walking-state) dataset, the C5.0 algorithm discriminated the stroke group and the control group with the highest accuracy (89%), the highest precision (88%), and maximum sensitivity (94%). In the functional motor task (working-state) dataset, the C5.0 algorithm discriminated the stroke group and the control group with the highest accuracy (84%) and the maximum sensitivity (89%). In the cognitive (reading-state) dataset, the C5.0 algorithm classified the stroke patients and the healthy adults with 85% accuracy (AUC: 0.86), the highest precision (83%), and the highest specificity (87%). Overall, SVM classified the task-independent dataset with 76% accuracy (AUC: 0.84). The Random Tree model predicted the stroke patients with the highest sensitivity (74%). The highest precision (69%) was achieved using logistic regression algorithms. The ROC performance curves of the machine-learning models for stroke prediction using resting, walking, working, reading, and task-independent entire datasets are demonstrated in Figure 8.

#### 3.2.2. Classification of the Resting State and Active State

As displayed in Table S4, we utilized the machine-learning algorithms to classify the resting state and the active (walking, working, and reading) states, regardless of the stroke group and the control group. The SVM model classified the resting and the active neural states with the highest accuracy (90%) and the highest AUC (0.84). Discriminant analysis algorithms showed the highest precision (94%), indicating the maximum portion of true positives. The neural network model predicted the active and resting class with perfect sensitivity (100%). SVM, logistic regression, and CHAID models performed the classification with nearly perfect sensitivities and high accuracies. The SVM model served the classification with the maximum Gini coefficient (0.69). The ROC performance curves of all models to predict the resting and active neural states are exhibited in Figure S1.

## 4. Discussion

In our study, we intended to characterize the electrical activity of the brain through EEG data of stroke patients and healthy adults during various tasks to understand the changes in neural states with the varied mental workload. EEG is a widely used non-invasive neuro-imaging technique. Continuous EEG monitoring can be applied for early prognostics [3]. Moreover, EEG is a potential tool for post-stroke physiology, functional outcomes, and cognitive decline [19]. Specific EEG band power is associated with the specific functional outcome of the brain and, in the case of ischemic stroke, is linked to the degree of neural damage in the lesion area of the brain. The outcome of this study also adds to our understanding of abnormal hemispheric asymmetry in homologous channel pairs, which represents the most prominent marker of the human awake resting-state EEG in the stroke-impaired brain. A comparative analysis of the proposed work and previous works is presented in Table 6.

The alpha power was significantly lower in the stroke population compared to the healthy adult population during resting, walking, and reading states. An exception was observed in the working condition, showing higher alpha activity during motor tasks such as working. As alpha frequencies are supposed to originate from cortical layers, cortical impairment due to stroke lesions may weaken alpha activity. Studies of combined EEG, MRI, CT research reflected alpha attenuation as a sign of brain injury [19,25]. Beta activity is higher in the stroke group than the control group in the working and cognitive tasks. Resting beta power shows a slightly opposite trend. Previous studies also have provided no reliable measures of post-stroke pathophysiology using beta activity [19,26].

Weaker theta activity was observed in the stroke group relative to that of the control group for all tasks. Although theta was criticized as an unreliable measure to predict post-stroke pathology, functional outcomes, and cognitive impairment [19], a few studies revealed theta activity as a prospective biomarker of post-stroke pathology and capable of discriminating between stroke patients and healthy controls [27], predicting functional outcomes [28], and cognitive deficits [29].

The slow-wave delta activity is considered as the most reliable prognostic measure relative to faster wave activity. Delta waves are supposed to derive in nerve cells in the thalamus and deep cortical layers. Delta activity may suggest hyperpolarization and curbing of cortical nerve cells, hampering neural activity. An increase in abnormal delta is often associated with brain injury (lesion) location [25]. Previous studies revealed higher delta power in the electrodes of the cortical positions in post-stroke recording [7,31,32]. In the current study, DAR showed a statistically significant difference between the stroke group and the healthy control group during the resting, motor, and cognitive tasks. In the stroke group, delta and theta have weak negative correlations in all mental states, whereas the weak negative correlation of delta-theta data exists among the healthy adults in all neurological states except the working condition. Delta-alpha data showed a weak positive correlation in the control group during the motor working phase. DAR was reported as the most consistent neural feature for the prognostics of ischemic stroke and post-stroke recovery [3,7,14,17,26]. The DTABR was considered as another effective predictor of neurological changes derived from ischemic stroke. Therefore, it revealed that DAR could be considered as a reliable predictive biomarker not only for the prognostics of stroke, but also for the post-stroke recovery. DTR is also observed as a potential marker of cognitive outcomes in the post-stroke phase [3,7]. Higher theta power was found to be associated with healthy cognitive outcomes [33].

The asymmetric measures of left–right cortical EEG bands possess prognostic importance in acute diseases [3]. The stroke-caused lesion weakens the cortical power over the scalp in the corresponding hemisphere and imbalances the symmetric behavior between the left and the right hemispheres. Beta and theta waves were significantly important asymmetric behavior between the stroke group and the control group in the resting state. Significant alpha and theta asymmetries were observed during the motor and cognitive tasks. Though delta asymmetry is a key feature for early prognostics of stroke [3], it showed no significant importance in post-stroke physiology. A higher pdBSI value indicates the lack of interhemispheric neuro-electrical balance due to the presence of the lesion caused by the ischemic stroke. The results of pdBSI showed a statistically significant difference between the stroke patient group and the control group in the cognitive tasks only. Earlier studies reported significantly different pdBSI in the stroke patient group relative to the healthy control group [3,22,27]. According to this study, no significant difference of pdBSI between the stroke and control group was observed in the resting state and motor tasks. This reveals that pdBSI may not be a discriminative tool for stroke patients in the near-recovery phase of post-stroke treatment. Lower pdBSI correlates with post-stroke recovery and the absence of lesion [34].

In this study, we conducted EEG only with six channels for understanding changes in EEG for neural impairment due to ischemic stroke. Although those cortical positions resemble other cortical positions, there are still chances of missing lesion locations. Therefore, the outcomes of the statistical investigation were limited to a few cortical electrodes. Similarly, the machine-learning model developed here is limited to only the central and parietal lobes with current parameterization.

## 5. Conclusions

We explored the neuro-electrical activity of stroke patients and healthy adults through EEG during resting, motor, and cognitive tasks. The spectral power features of alpha, theta, and delta waves were revealed as discriminative factors classifying the stroke patients and the healthy adults in the motor and cognitive states. DAR and DTR were biomarkers for the stroke and the healthy control group during the resting, motor, and cognitive tasks. Machine-learning algorithms were also utilized to classify stroke patients and healthy adults in different mental states. This study will be helpful in the management of post-stroke treatment and post-stroke recovery.

## Figures and Tables

**Table 1:** Results of the classification performance of different machine-learning models using the resting dataset.

Model	Accuracy	Sensitivity	Specificity	Precision	Negative Predictive Value	AUC	Gini
SVM	0.71	0.45	0.87	0.70	0.71	0.77	0.54
Random Tree	0.73	0.48	0.89	0.74	0.72	0.78	0.56
Logistic Regression	0.70	0.54	0.80	0.64	0.72	0.74	0.48
C5.0	0.78	0.60	0.90	0.79	0.77	0.84	0.69

**Table 2:** Results of the classification performance of different machine-learning models using the walking dataset.

Model	Accuracy	Sensitivity	Specificity	Precision	Negative Predictive Value	AUC	Gini
SVM	0.86	0.90	0.81	0.85	0.87	0.92	0.84
Random Tree	0.82	0.86	0.77	0.82	0.82	0.89	0.78
Logistic Regression	0.81	0.84	0.78	0.82	0.81	0.87	0.73
C5.0	0.89	0.94	0.84	0.88	0.92	0.90	0.79

**Table 3:** Results of the classification performance of different machine-learning models using the working dataset.

Model	Accuracy	Sensitivity	Specificity	Precision	Negative Predictive Value	AUC	Gini
SVM	0.80	0.82	0.77	0.82	0.78	0.88	0.77
Random Tree	0.78	0.79	0.78	0.81	0.74	0.85	0.70
Logistic Regression	0.75	0.79	0.69	0.76	0.73	0.84	0.67
C5.0	0.84	0.89	0.79	0.84	0.85	0.87	0.73

**Table 4:** Results of the classification performance of different machine-learning models using the cogitative reading dataset.

Model	Accuracy	Sensitivity	Specificity	Precision	Negative Predictive Value	AUC	Gini
SVM	0.78	0.71	0.84	0.78	0.79	0.86	0.72
Random Tree	0.75	0.62	0.85	0.76	0.74	0.81	0.62
Logistic Regression	0.71	0.61	0.79	0.70	0.72	0.78	0.56
C5.0	0.85	0.82	0.87	0.83	0.86	0.86	0.71

**Table 5:** Results of the classification performance of different machine-learning models using the task-independent entire dataset.

Model	Accuracy	Sensitivity	Specificity	Precision	Negative Predictive Value	AUC	Gini
SVM	0.76	0.73	0.79	0.77	0.76	0.84	0.69
Random Tree	0.70	0.74	0.66	0.68	0.73	0.78	0.56
Logistic Regression	0.69	0.67	0.71	0.69	0.70	0.76	0.53
C5.0	0.70	0.71	0.69	0.69	0.71	0.74	0.49

**Table 6:** Comparative study of methodologies and results between proposed work and previous works.

Study	EEG Time after Stroke Onset	Study Sample	EEG Features of Neurological Outcome	Main Findings
Finnigan et al. [26]	46–52 h	Thirteen stroke patients	DAR; relative alpha; DTABR	DAR was correlated with 30-day NIHSS
Sheorajpanday et al. [30]	<6 months	One hundred and ten stroke patients	DTABR, pdBSI	DTABR and pdBSI were correlated with the Modified Rankin scale and disability after six months after stroke
Finnigan et al. [16]	<24 h	Twenty-eight healthy control and eighteen stroke patients	QEEG indices (power of delta, theta, alpha, and/or beta bands), DAR	EEG power of delta, theta, alpha, and/or beta bands differed highly significantly between acute stroke (IS) and control; DAR demonstrated the highest accuracy for discriminating between acute IS patients and controls.
Aminov et al. [7]	<72 h	Twenty-four stroke patients	DTR, RP theta, RP delta, and DAR	DTR, RP theta, RP delta, and DAR were correlated with 90-day MoCA scores.
Proposed work	<3 months	Forty-eight stroke patients and seventy-five healthy adults	EEG indices (power of delta, theta, alpha bands), DAR, DTR	EEG indices (power of delta, theta, alpha bands), DAR, DTR were discriminative biomarkers in the resting, motor, and cognitive reading task.

## Supplementary Materials

The following are available online at https://www.mdpi.com/article/10.3390/brainsci11070900/s1, Table S1: Statistical analysis of the EEG spectral features for the Control group and the Stroke group during the resting, motor, and cognitive tasks. Table S2: Statistical analysis of the asymmetry of spectral components for the Control group and the Stroke group. Table S3: Statistical analysis of the DTR, DTABR, and DAR for the Control group and the Stroke group. Table S4: Results of the performance of different Machine learning Models for classification of the resting and the active states. Figure S1: Receiver Operating Characteristic ROC curves of five different machine-learning models. Figure S2: The scatterplot and the regression line of delta power with theta power and alpha power.
