# Supplementary material for: Quantitative Evaluation of Task-Induced Neurological Outcome after Stroke
Source: Brain Sci. 2021 Jul 7;11(7):900. doi: 10.3390/brainsci11070900 (PMC8307254; doi:10.3390/brainsci11070900)
Supplement: Supplementary file 1 [file brainsci-11-00900-s001.zip › brainsci-1237411-supplementary.pdf]

Table S1. Statistical analysis of the EEG spectral features for the Control group and the Stroke group during the resting, motor, and cognitive tasks. Resting state is considered as the baseline. * indicates p < 0.05.

EEG Features	Scenario	Mean Value		Standard Deviation		Change from Baseline (%)			p-value
		Control	Stroke	Control	Stroke	Control	Stroke	Difference	
Alpha (Relative Power)	Baseline (Resting)	0.51	0.47	0.21	0.22	-	-	-	0.204
	Walking (Motor)	0.47	0.43	0.20	0.28	-0.05	-0.13	0.08	0.001*
	Working (Motor)	0.39	0.42	0.14	0.20	-0.21	-0.14	-0.07	0.0001*
	Reading (Cognitive)	0.49	0.48	0.19	0.27	-0.004	-0.016	0.012	0.0001*
Beta (Relative Power)	Baseline (Resting)	0.78	0.74	0.47	0.47	-	-	-	0.202
	Walking (Motor)	0.50	0.53	0.38	0.42	-0.35	-0.31	-0.04	0.055
	Working (Motor)	0.52	0.69	0.30	0.39	-0.32	-0.10	-0.22	0.0001*
	Reading (Cognitive)	0.74	0.75	0.42	0.56	-0.03	-0.02	-0.01	0.002*
Theta (Relative Power)	Baseline (Resting)	0.77	0.73	0.27	0.29	-	-	-	0.608
	Walking (Motor)	1.07	0.83	0.41	0.29	0.42	0.09	0.33	0.0001*
	Working (Motor)	0.88	0.73	0.35	0.29	0.16	-0.04	0.20	0.003*
	Reading (Cognitive)	0.84	0.79	0.15	0.28	0.11	0.04	0.07	0.022*
Delta (Relative Power)	Baseline (Resting)	3.60	3.70	0.90	0.98	-	-	-	0.119
	Walking (Motor)	3.74	3.97	0.71	0.95	0.03	0.09	-0.06	0.0001*
	Working (Motor)	3.74	3.56	0.72	0.99	0.03	-0.02	0.05	0.0001*
	Reading (Cognitive)	3.65	3.64	0.85	0.97	0.002	0.001	0.001	0.001*
Gamma (Relative Power)	Baseline (Resting)	0.33	0.36	0.33	0.31	-	-	-	0.584
	Walking (Motor)	0.22	0.25	0.30	0.31	-0.36	-0.27	-0.09	0.163
	Working (Motor)	0.48	0.60	0.48	0.71	0.39	0.76	-0.37	0.0001*
	Reading (Cognitive)	0.28	0.34	0.21	0.29	-0.18	-0.02	-0.16	0.0001*

Table S2. Statistical analysis of the asymmetry of spectral components for the Control group and the Stroke group during the resting, motor and cognitive tasks. Resting state is considered as the baseline. * indicates p < 0.05.

EEG Features	Scenario	Mean Value		Standard Deviation		p-value
		Control	Stroke	Control	Stroke	
Alpha Asymmetry	Resting	0.13	0.12	0.083	0.081	0.726
	Walking (Motor)	0.109	0.112	0.07	0.09	0.033*
	Working (Motor)	0.09	0.10	0.06	0.07	0.03*
	Reading (Cognitive)	0.10	0.11	0.04	0.07	0.0001*
Beta Asymmetry	Resting	0.19	0.17	0.11	0.09	0.014*
	Walking (Motor)	0.16	0.15	0.10	0.10	0.496
	Working (Motor)	0.15	0.09	0.09	0.09	0.57
	Reading (Cognitive)	0.17	0.16	0.09	0.09	0.460
Theta Asymmetry	Resting	0.09	0.11	0.05	0.08	0.0001*
	Walking (Motor)	0.07	0.08	0.05	0.07	0.002*
	Working (Motor)	0.07	0.08	0.05	0.06	0.31
	Reading (Cognitive)	0.08	0.09	0.03	0.07	0.0001*
Delta Asymmetry	Resting	0.08	0.07	0.06	0.06	0.738
	Walking (Motor)	0.06	0.06	0.05	0.06	0.172
	Working (Motor)	0.06	0.07	0.05	0.05	0.79
	Reading (Cognitive)	0.065	0.072	0.05	0.06	0.087
Gamma Asymmetry	Resting	0.24	0.21	0.13	0.12	0.191
	Walking (Motor)	0.21	0.19	0.12	0.12	0.266
	Working (Motor)	0.20	0.19	0.12	0.11	0.76
	Reading (Cognitive)	0.21	0.20	0.11	0.11	0.99
pdBSI	Resting	0.12	0.11	0.06	0.06	0.487
	Walking (Motor)	0.10	0.10	0.06	0.07	0.318
	Working (Motor)	0.09	0.10	0.05	0.05	0.29
	Reading (Cognitive)	0.10	0.11	0.04	0.07	0.001*

Table S3. Statistical analysis of the DTR, DTABR, and DAR for the Control group and the Stroke group during the resting, motor and cognitive tasks. Resting state is considered as the baseline. * indicates p < 0.05.

EEG Features	Scenario	Mean Value		Standard Deviation		p-value
		Control	Stroke	Control	Stroke	
DAR	Resting	11.10	13.51	10.57	17.54	0.039*
	Walking (Motor)	11.14	14.65	8.62	18.48	0.0001*
	Working (Motor)	11.98	13.17	8.30	13.17	0.034*
	Reading (Cognitive)	10.62	12.36	4.82	17.46	0.0001*
DTR	Resting	5.83	6.72	4.04	9.67	0.007*
	Walking (Motor)	4.46	6.05	3.12	6.33	0.006*
	Working (Motor)	5.22	3.63	3.63	4.43	0.09
	Reading (Cognitive)	5.13	5.86	3.05	5.81	0.0001*
DTABR	Resting	5.69	6.73	7.65	8.90	0.138
	Walking (Motor)	7.33	8.80	4.92	9.95	0.0001*
	Working (Motor)	6.78	5.90	4.33	5.62	0.03*
	Reading (Cognitive)	5.67	6.50	4.80	8.70	0.0001*

Hussain, Iqram, Park, Se Jin. Quantitative Evaluation of Task-induced Neurological Outcome after Stroke. *Brain Sci.* **2021**, *11*, x.

Table S4. Results of the performance of different Machine learning Models for classification of the resting and the active (walking, working, and reading) states.

Model	Accuracy	Sensitivity	Specificity	Precision	Negative Predictive Value	AUC	Gini
**SVM**	0.90	0.98	0.351	0.92	0.68	0.84	0.69
**Logistic Regression**	0.88	0.99	0.092	0.89	0.47	0.74	0.47
**Neural Network**	0.88	1.00	0.005	0.88	0.29	0.74	0.41
**CHAID**	0.88	0.99	0.104	0.89	0.53	0.77	0.54
**Discriminant Analysis**	0.70	0.71	0.671	0.94	0.24	0.73	0.47

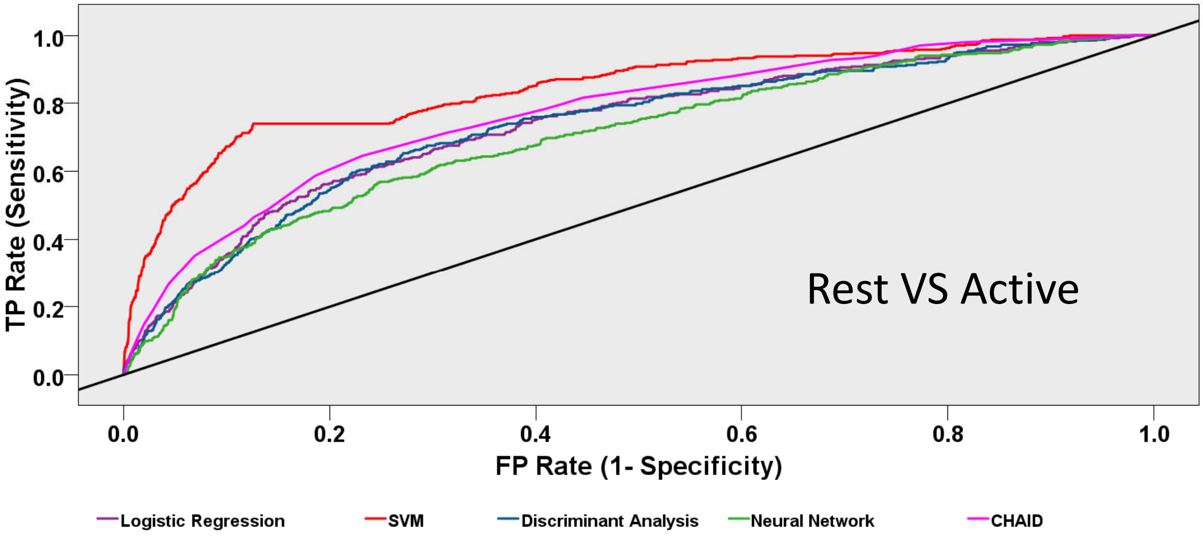

Figure S1. Receiver Operating Characteristic (ROC) curves of five different machine-learning models (Support Vector Machine, Logistic Regression, Discriminant analysis, Neural Network and CHAID decision tree) for classification of the resting and the active (walking, working, and reading) states. SVM classified the resting and the active dataset with the highest AUC (0.84) and highest accuracy (ACC: 90%). The diagonal black line is the reference line.

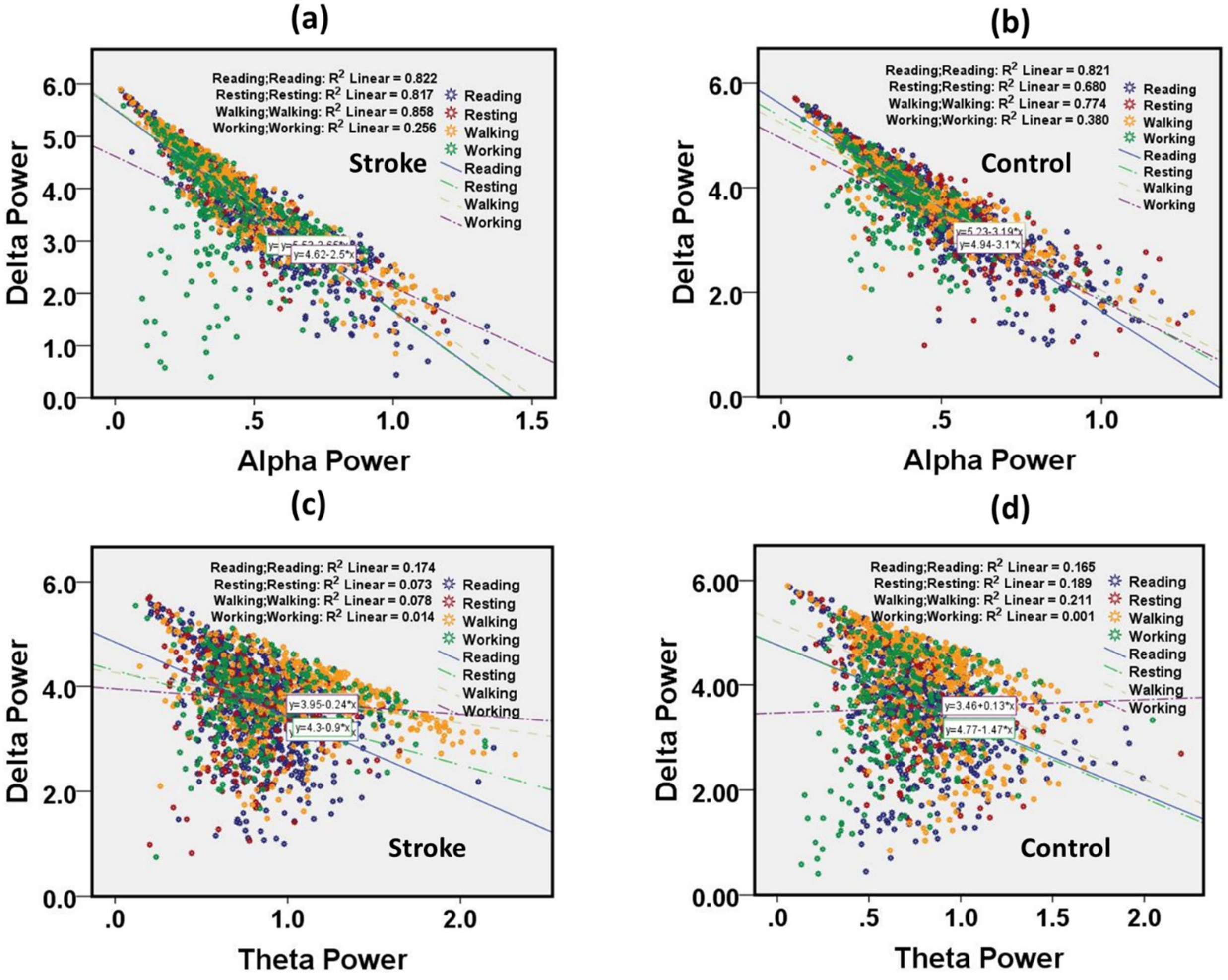

Figure S2. The scatterplot and the regression line of delta power with theta power and alpha power to demonstrate the correlation of delta power with theta and alpha power during varied mental workloads (Resting, Walking, Working, Reading tasks). (a) the correlation of delta power with alpha power for stroke group (b) the correlation of delta power with alpha power for control group (c) the correlation of delta power with theta power for stroke group (d) the correlation of delta power with theta power for control group. R^2 or r^2 (R squared) = Coefficient of determination.
